# Microstructure-Based Computational Analysis of Deformation Stages of Rock-like Sandy-Cement Samples in Uniaxial Compression

**DOI:** 10.3390/ma16010024

**Published:** 2022-12-21

**Authors:** Mikhail O. Eremin, Valentina A. Zimina, Aleksey S. Kulkov, Yurii P. Stefanov

**Affiliations:** Institute of Strength Physics and Materials Science of Siberian Branch of Russian Academy of Sciences, 8/2 Akademicheskii Pr., 634055 Tomsk, Russia

**Keywords:** experimental study, numerical modeling, rock-like material, sandy cement, fracture, damage accumulation, threshold stresses, constitutive equation, loading diagrams, dilatancy, acoustic emission

## Abstract

This work presents a new finite-difference continuum damage mechanics approach for assessment of threshold stresses based on the mechanical response of a representative volume element of a sandy-cement rock-like material. An original experimental study allows validating the mathematical model. A new modification of the damage accumulation kinetic equation is proposed. Several approaches based on acoustic emission, instantaneous Poisson’s ratio and reversal point method are employed to determine the threshold stresses. Relying on the numerical modeling of deformation and failure of model samples, the threshold stresses and the deformation stages are determined. The model predicts the crack initiation stress threshold with less than 10% error. The model prediction of the crack damage stress threshold corresponds to the upper boundary of the experimental range. The model predicts the peak stress threshold with less than 0.2% error in comparison with the average experimental peak stress. The results of numerical modeling are shown to correlate well with the available experimental and literature data and sufficiently complement them.

## 1. Introduction

Recent advances in rock mechanics have revealed the threshold stresses distinguishing the deformation stages of different rocks. It was found out that crack initiation, crack damage and peak stresses are useful for rock pressure control in tunneling and underground technologies.

Based on the literature data analysis, the stages of uniaxial deformation of rocks have been extensively investigated in numerous experimental studies, for instance [1,2,3,4,5,6,7]. In these works, an evaluation of threshold stresses was carried out by means of complete stress-strain curves of loaded rocks, acoustic emission series, instantaneous Poisson’s ratio functions, tangential modulus, and axial and volumetric stiffness. The thresholds are related to the corresponding stages of the deformation process. The following threshold stresses are generally distinguished: the microcrack closure stress, the crack initiation stress, the crack damage stress, the peak stress. The microcrack closure stress indicating the end of the initial nonlinear concave part of the loading diagram is seldom applied in practice, although the corresponding microcrack closure strain allows estimating the microcrack density and texture in rocks [8,9]. The other thresholds are more significant from the point of their specific engineering applications.

For instance, Nicksiar and Martin [7] presented a comprehensive study on the crack initiation stress in igneous and sedimentary rocks. Statistical results reveal the ratio of the crack initiation stress to uniaxial compression strength (UCS) ≈ 0.45. Martin and Christiansson [10] argued that crack initiation stress only slightly underestimates the in-situ strength when comparing the in-situ spalling strength of the circular opening with the crack initiation stress of a laboratory tested Lac du Bonnet granite sample. Similar values of the spalling stress-to-compressive strength ratios of the rock massive at the Baihetan exploratory tunnel were also reported by Jiang et al. [11]. Esterhuizen et al. [12] performed a study of pillar failure in hard rock mines; the authors argue that according to the in-situ measurements, the pillars tend to failure under the stress an order of magnitude lower than the UCS of the laboratory samples.

The crack damage stress indicative of the onset of an unstable crack propagation stage was studied in many works, e.g., [3,6,13,14]. Cai et al. [3] replaced the UCS of an intact rock with that of the jointed rock mass and obtained the threshold initiation and damage criteria. It was shown that the thresholds assist in assessing the rock mass integrity. Martin and Chandler [6] argued that upon reaching the crack damage stress the fracture process becomes self-sustaining, which is indicative of a long-term strength of rocks. Xue et al. [13] reported a high impact of porosity on the ratio of the crack damage stress, although the origin of rocks has hardly any influence. The crack damage stress was studied by Zhao et al. [14] based on the acoustic emission and complete stress-strain curve. The authors state that their results are applicable to prediction of the underground excavation response and might be easily implemented as a ready-to-use damage criterion in any numerical codes.

In the field of mathematical modeling, several approaches have been successfully applied to studying the stages of deformation and fracture of rocks under uniaxial loading, e.g., [15,16,17,18]. Ghazvinian et al. [15] applied the 3DEC to investigating the different aspects of rock behavior with an explicit consideration of textural features using the Voronoi tessellation. The model reproduces the effective mechanical characteristics of loaded samples and the features of failure. The bonded-particle model was proposed by Potyondy and Cundall [16] and applied to modeling the Lac du Bonnet granite failure as a part of the particle flow code. The bonded-block model was represented by Sinha and Walton [17], the authors proposed an inverse tangent lateral stiffness technique for evaluation of the crack initiation stress and normalized number of crack techniques for evaluation of the crack damage stress. Zhou et al. [18] considered a random distribution of microcracks in a rock volume and proposed a microcrack constitutive damage model considering the fracture and damage mechanics and the reduction in Young’s modulus of a material in the course of damage accumulation. Liu et al. [19] studied the fracture evolution of crystalline basalt both under uniaxial and triaxial loading. It was shown that an increase in the confinement stress changes the dominant failure mechanism. Wang et al. [20] studied the features of rock mass failure. The uniaxial compressive strength and deformation characteristic exhibit a non-monotonic U-shaped dependence with the maximums at the 0° and 90° inclination angles as reported by the study. Zhou et al. [21] employed the COMSOL 3D finite element package to describe the cracks coalescence patterns of the heterogeneous Beishan granite containing a random initial distribution of micro-defects under uniaxial compression and Brazilian test. Wang et al. [22] applied the conjugated bond-based peridynamics to modeling the crack propagation and coalescence in rocks under uniaxial compression. Zhao [23] analyzed the crack patterns of the Fangshan marble and proposed a constitutive damage model describing fairly good the experimental pre-peak complete stress-strain curve. Xie et al. [24] applied a micro-mechanics-based elastoplastic damage constitutive model to study the response of the fresh and weathered diabase samples under triaxial compression. Yuan et al. [25] applied an elastoplastic damage constitutive model to compression of rock-like materials with consideration of cracks interaction. A descending portion of the loading diagram is observed when the scalar damage parameter exceeds ≈0.1. Zhang et al. [26] proposed a constitutive model of the weathered granite deformation based on the AE series to describe an integral damage of the sample.

According to the literature review, there are few articles accurately assessing the threshold stresses and the corresponding deformation stages based on the 3D numerical modeling of uniaxial compression due to a general complexity of the problem. Moreover, rocks with a complex mineral content are inherently heterogeneous. This brings difficulties in distinguishing the influence of the individual minerals on the loading response of the samples. Numerical modeling faces the same problems when it comes to interpreting experimental observations. One needs to describe properly the constitutive equations for all constituents of a natural composite material. For this reason, it is important for the experimental samples to be prepared from the most homogeneous rock formations. Since the latter are seldom found in nature, one can calibrate the numerical models against the data obtained from laboratory experiments with the artificial rock-like materials. The practical engineering significance of such assessments is due to the following: the regularities of mechanical behavior of pillars in room-and-pillar and other types of mining have much in common with the mechanical behavior of the laboratory samples subjected to uniaxial compression.

In this work, we prepared sandy-cement rock-like material samples for an experimental study. The samples have a relatively homogeneous structure and a very low porosity (less than 2%). They are subjected to a uniaxial compression test with registration of the lateral strain, and the complete stress-strain curves of material were obtained. The experimental results are further used as a guide for the mathematical model calibration. Special attention is paid to the stages of deformation process which are distinguished based on the simulated acoustic emission pulses, the instantaneous Poisson’s ratio, and the reversal point method. Computer modeling of the uniaxial compression test is carried out in the framework of the three-dimensional finite-difference method (FDM). The Representative Volume Element (RVE) with an explicit consideration of the porous structure (spherical pores) is designed for the study. We propose a new finite-difference continuum damage mechanics approach for assessment of threshold stresses based on the mechanical response of a representative volume element of a sandy-cement rock-like material.

## 2. Material

**Figure A1:**
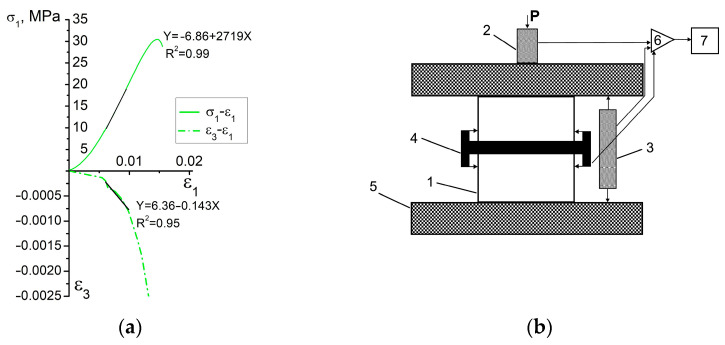
Method for determination of Young’s modulus and Poisson’s ratio of sample (**a**), schematic experimental setup, 1—sample, 2—force gauge, 3—vertical strain gauge, 4—lateral strain gauge, 5—loading plates, 6—3-channel amplifier, 7—analog to digital converter (**b**).

**Figure A2:**
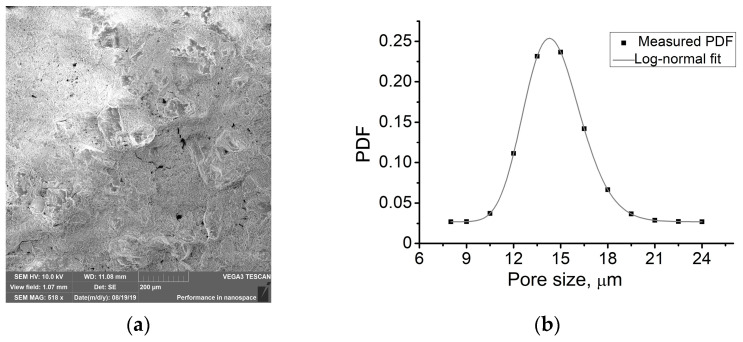
SEM pattern of the etched sample surface (**a**), probability density function of pore size distribution (**b**).

For the manufacture of initial sample, Portland cement grade M500 and river sand were used. The dust fraction of sand was removed by screening. The sand-cement mixture was mixed with water at a ratio of 0.58 and left in the cassette on the vibrating table for 30 min. The ratio of volume fractions of cement and sand was 1:2. Sample was left for 28 days for solidification. The details of the method employed are discussed elsewhere [27]. The density of material was determined as the mass-to-volume ratio of sample, and the pore volume was disregarded. Young’s modulus and Poisson’s ratio of the material are determined from the elastic part of complete stress-strain curve (see Figure A1a in Appendix A as an example). Based on the SEM pattern of the etched sample surface (see Figure A2a in Appendix A), the total porosity of the material is ≈2%, the pore size distribution is illustrated in Figure A2b. Some of these data are further directly transferred as the model parameters (ρ, E, and ν), the other experimental parameters are used to validate the model parameters. The design of the proposed structural model is discussed in Appendix A.

## 3. Experimental Study

A total of 10 sandy-cement rock-like parallelogram samples were cut by a diamond disc from a larger sample. The laboratory loading of the samples was performed using an Instron 1185 universal testing machine in the deformation-controlled mode with the loading rate of 0.05 mm/min. The longitudinal strain was recorded by means of the Instron firmware, while the lateral strain was recorded simultaneously by the gauge installed around the sample. Both longitudinal and lateral strains were determined using engineering strain notation. Stresses were determined as the force value divided by the sample cross-section area. Schematic experimental setup is illustrated in Figure A1b in Appendix A.

**Figure 1:**
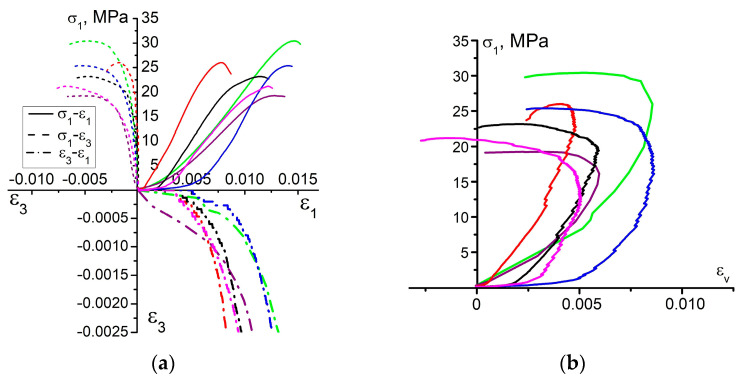
Complete stress-strain diagram of loaded samples: (**a**) curves of dependence *σ*_1_ − *ε*_1_, *σ*_1_ − *ε*_3_, and *ε*_1_ − *ε*_3_; (**b**) curves of dependence *σ*_1_ − *ε*_*v*_. Different colors correspond to different tested samples.

The resulting complete stress-strain diagrams of 6 samples are illustrated in Figure 1a. The other samples were disregarded due to a sufficient deviation from the presented group of samples. Judging by the results, the convergence of the loading curves is poor, although the samples were prepared in the laboratory, carefully controlled conditions. This is typically observed in almost all experimental studies of uniaxial compression of rocks or rock-like materials. The Young’s modulus and the UCS value of the tested samples vary in a vast range (3.14 ± 1 GPa and 24.3 ± 5 MPa, respectively), which is apparently due to the pores in the bulk of material. Pores cause stress concentration and become the regions of cracks nucleation. It is well known that the residual porosity noticeably influences the physical-mechanical parameters of the materials [28,29,30]. An increase in porosity causes a rather noticeable decrease in Young’s modulus and UCS. Therefore, the scatter of properties is quite a common phenomenon, though undesirable.

According to the classification of deformation stages proposed by Martin and Chandler [6], the loaded samples exhibit all stages of the deformation process. The initial stage of loading has a non-linear concave segment in the $\sigma_{1}-\varepsilon_{1}$ curve. This stage is generally treated as the microcrack and/or micropore closure causing a gradual increase in the tangential modulus. Notably, in this stage of deformation the lateral strain remains approximately zero. Thus, the deformation occurs to some extent only in the loading direction. This proves the microcrack closure being a possible mechanism driving the tangential modulus change. The ratio $\frac{\sigma_{cc}}{\sigma_{p}}$ for the tested samples might be as high as 0.23, which is indicative of a quite long stage of the micropores closure.

The linear dependence of the $\sigma_{1}-\varepsilon_{1}$ curve is observed in the next stage of deformation. Judging by the $\sigma_{1}-\varepsilon_{3}$ curves, the elastic stage is not long. A departure from linearity in these curves is indicative of a crack initiation process which points out the onset of the next stage. The newly formed cracks exhibit a stable growth after the $\sigma_{ci}$ threshold is exceeded. Based on the analysis, the ratio of the crack initiation-to-peak stress lies in the range of ≈0.45–0.63. This range is quite consistent with the data reported in [5,6].

To determine the crack damage threshold $\sigma_{cd}$, we plot the dependence of axial stress $\sigma_{1}$ on volumetric strain $\varepsilon_{v}$ using expression $\varepsilon_{v}\approx \varepsilon_{1}+2\varepsilon_{3}$ (Figure 1b). This curve is of great importance for engineering. So, as reported by Bieniawski [1], the reversal point, wherein the derivative of $\sigma_{1}-\varepsilon_{v}$ curve changes its sign, is taken as a crack damage threshold $\sigma_{cd}$. Upon reaching the $\sigma_{cd}$ threshold, cracks become unstable. For this reason, the onset of unstable crack growth is generally referred to as the long-term strength of rocks [1]. Interestingly, the conclusions drawn in [1] mostly relied on the data of hard rock testing—igneous rocks and metamorphic sedimentary rocks. These rocks generally have greater strengths and more brittle character of failure in uniaxial compression due to their sufficiently later diagenesis.

**Figure 2:**
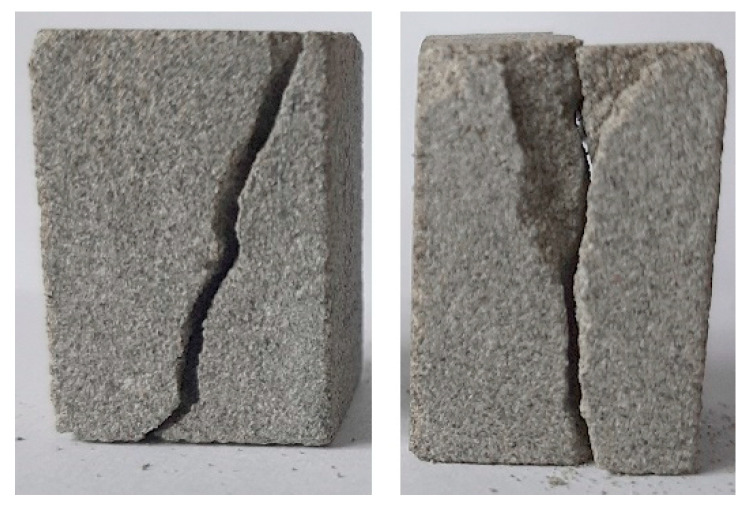
Typical failure patterns of samples.

Finally, when the peak stress $\sigma_{p}$ is reached, the stress-strain curve is observed to descend. In this stage, the final crack pattern is formed. Samples are generally divided into several pieces of different sizes. Figure 2 illustrates several failure patterns of the tested samples. It can be noted that the major driving failure mechanisms are the ≈70°-inclined shear and sub-vertical tensile cracks.

The experimental data are summarized in Table 1.

## 4. Mathematical Formulation of the Boundary Value Problem

To provide a better insight into the fracture process of the material in question, the finite-difference continuum damage mechanics approach is employed. The system of equations includes the laws of mass Equation (1) and momentum Equation (2) conservation. The stress tensor is symmetric $\sigma_{ij}=\sigma_{ji}$, which also satisfies the law of the angular momentum conservation.

(1) $$\rho_{0}V_{0}=\rho V$$

(2) $$\rho {\dot{v}}_{i}=\frac{\partial \sigma_{ij}}{\partial x_{j}}$$

The system also includes the geometrical relations for the strain rate (Equation (3)) and vorticity tensors (Equation (4))

(3) $$2{\dot{\varepsilon }}_{ij}=\frac{\partial v_{i}}{\partial x_{j}}+\frac{\partial v_{j}}{\partial x_{i}}$$

(4) $$2{\dot{\omega }}_{ij}=\frac{\partial v_{i}}{\partial x_{j}}-\frac{\partial v_{j}}{\partial x_{i}}$$

### 4.1. Constitutive Equations

The stresses are calculated according to the relations of a hypo-elastic medium with separation of the volumetric and deviatoric parts of stress tensor. Equations (5) and (6) allow for calculating the increments of the volumetric and deviatoric parts, respectively. The corotational Jaumann derivative is applied in Equation (6) to subtract the rotation of an element as a whole, which does not influence the values of deviatoric stresses.

(5) $$\dot{P}=-K(\frac{\dot{V}}{V}-{\dot{\varepsilon }}^{P})$$

(6) $${\dot{S}}_{ij}+S_{ik}{\dot{\omega }}_{kj}-S_{kj}{\dot{\omega }}_{ik}=2\mu [{\dot{\varepsilon }}_{ij}-\frac{1}{3}(\frac{\dot{V}}{V}-{\dot{\varepsilon }}^{P})\delta_{ij}-{\dot{\varepsilon }}_{ij}^{P}]$$

A slightly modified version of the original Drucker-Prager (DP) criterion [31] is employed as part of the constitutive response to describe the inelastic deformation of samples. An evolution of cohesion depends on the equivalent inelastic strain according to the law proposed elsewhere [32] and on the accumulated damage D.

(7) $$f(\sigma_{ij},D)=-\alpha P+\tau -Y$$

(8) $$Y=f(\gamma^{P})=Y_{0}[1+h(2\frac{\gamma^{P}}{\gamma^{*}}-{\left(\frac{\gamma^{P}}{\gamma^{*}}\right)}^{2})](1-D)$$

In Equations (7) and (8), α and Y are the material constants associated with rock cohesion C and internal friction angle ϕ [31], $\tau =\sqrt{J_{2}}=\sqrt{\frac{1}{2}S_{ij}S_{ij}}$. For simplicity, we will further refer to α as the internal friction factor and Y as the cohesion. The cohesion evolution in the course of inelastic strain accumulation is discussed in detail in Appendix A.

The inelastic strain rate tensor components are defined according to the plastic potential (Equation (9)) from the theory of plasticity (Equation (10)). The non-associated flow rule is employed.

(9) $$g(\sigma_{ij})=\tau -\Lambda P+const$$

(10) $$d\varepsilon_{ij}^{P}=d\lambda \frac{\partial g(\sigma_{ij})}{\partial \sigma_{ij}}$$

As a result of derivation, we obtain the following equation for the increments of the inelastic strain tensor components (Equation (11)):

(11) $$d\varepsilon_{ij}^{P}=d\lambda (\frac{S_{ij}}{2\tau }+\frac{\Lambda }{3}\delta_{ij})$$

Multiplier dλ is defined in the calculations when Equation (7) is satisfied. The procedure of stress correction due to inelastic deformation is described elsewhere [33].

The theory of continuum damage mechanics (CDM) is applied in this work to describe the rock fracture process. Here, we propose a new modification of the kinetic equation for the damage measure time derivative $D(\sigma_{C},t)$ (Equation (12)). The shape of the right side of equation conforms to the energy necessary to transfer crystal from initial state to the state of local shear [34].

(12) $$\frac{dD}{dt}=\frac{\sigma_{C}^{2}}{t_{*}},$$

where in $\sigma_{C}=\tau /(Y+\alpha P)$ is the Coulomb stress, and $t_{*}$ is the model parameter controlling the rate of damage accumulation having the physical meaning of the characteristic time of the fracture incubation process, it was put equal to 10^2^ due to following: all tested samples were loaded during ≈200 s and average crack initiation stress to peak stress is 0.53, therefore, 10^2^ s is the approximate fracture incubation process time.

**Figure 3:**
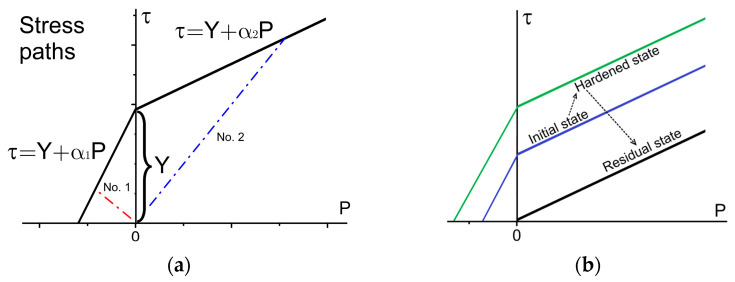
Yield/damage surface and possible stress paths in elastic stage of deformation (**a**), schematic representation of the yield/damage surface evolution (**b**).

When the fracture criterion is met in a particular material point, the stress-strain evolution transits to the post-peak behavior (see Figure 3b for illustration of yield surface in residual state); its description is also very important for modeling the macroscopic response of the material. We use the following post-peak behavior in the local point:

1. if the hydrostatic pressure P< 0 then all stress tensor components are nullified;
2. if the hydrostatic pressure P> 0 then the material in the local point continues resisting the residual shear strength τ=αP (see Figure 3b for residual state yield strength).

An explanation of other parameters is provided in Appendix A.

### 4.2. Validation of Other Model Parameters against the Experimental Data

Based on a comprehensive review of the intact rock behavior [5], the yield strength of rocks generally has a parabolic shape limited by the tensile cut-off in the region of the negative minor principal stress. Therefore, it is impossible to describe the entire rock behavior with a single-slope envelope. In contrast to the linear yield envelope, a parabolic envelope is more complex in terms of deriving and obtaining the inelastic strain rate tensor components. In this work, we propose a piece-wise linear yield criterion with different slopes for negative and positive semi-spaces of the 2D Haigh-Westergaard-like stress space (axis τ is the von Mises stress, axis P is the hydrostatic pressure, see Figure 3). Parameters α and Y determine the yield surface. The values of these parameters are calculated according to the method described in Appendix A. Determination of the dilatancy factor Λ dependence on the inelastic volumetric strain is also discussed in Appendix A.

The possible loading histories of the elastic deformation stage in the model are schematically illustrated in Figure 3a with red and blue dashed-dotted lines. In the case of stress paths No. 1 and 2, inelastic deformation of a material point initiates when the criterion (3.1) (initial state) is met at the negative and positive semi-spaces, respectively. Schematic representation of the yield/damage surface evolution in the course of inelastic deformation and damage accumulation is illustrated in Figure 3b.

The model parameters are summarized in Table 2.

### 4.3. Boundary and Initial Conditions

The following boundary conditions are applied to the computational domain:

(i) the velocity vector component $v_{z}$ is assigned to the nodes belonging to the opposite Z-plane edges—$v_{z}=v(t)$ and $v_{z}=-v(t)$, respectively, tangential sliding of nodes is restricted;
(ii) the free-of-stress surface condition is maintained at all other facets of the sample and within pores.

The initial state of the sample corresponds to zero values of all stress-strain state parameters. The technique of slow loading was utilized to minimize the influence of the acceleration term in Equation (2). When determining the function v(t), the time of the loading velocity increase corresponds to more than 20 runs of an elastic P-wave through the entire computational domain. The latter also indicates the limitation of presented model—we focus only on quasi-static strain rates and do not consider dynamic loading of rocks which is known to produce the strain-rate sensitivity. To solve the boundary value problem, we used the finite-difference method proposed and exhaustively discussed by M.L. Wilkins [35].

## 5. Results and Discussion

### 5.1. Convergence of the Numerical Solution

**Figure 4:**
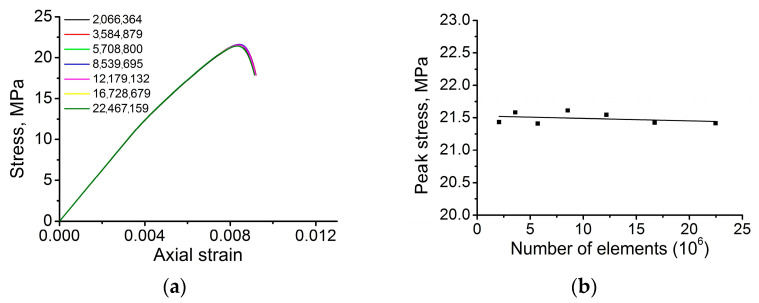
Verification of the numerical solution convergence: (**a**) stress-strain curves for models with different number of mesh elements, (**b**) peak stress of obtained stress-strain curves against the number of mesh elements.

**Figure 5:**
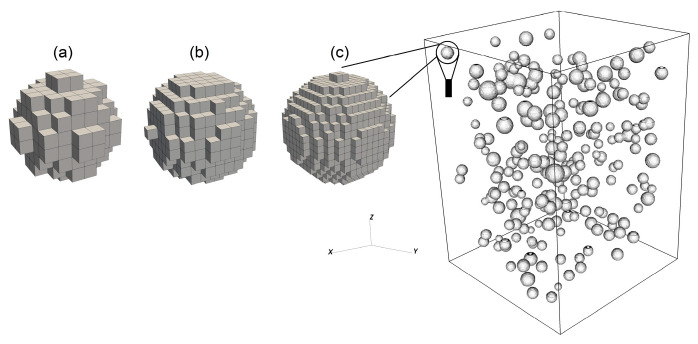
Description of a phase boundary using different meshes: (**a**) coarse (159 × 114 × 114 elements), (**b**) medium (255 × 183 × 183 elements), (**c**) fine (351 × 253 × 253 elements).

The mathematical model presented above contains the local fracture criterion complemented with the non-associated plastic flow rule. This combination is detrimental to numerical solution stability. The local criteria are known to produce spurious strain localizations, meanwhile the use of the non-associated plastic flow rule makes the model unstable due to violation of the Drucker postulate [36]. For this reason, the verification of the numerical solution convergence is a necessary step of numerical modeling. Herein, we verify the numerical solution convergence based on the curve of dependence of $\sigma_{zz}$ stress tensor component averaged over the entire volume of the computational domain versus the number of mesh elements. Figure 4 illustrates the results of the convergence test. A magnified inset of a near-peak region (Figure 4a) of the stress-strain curves obtained for models with different numbers of mesh elements suggests that the curves are almost identical. This is also supported by Figure 4b where the average peak stress is plotted against the number of mesh elements. The difference in the peak stress values does not exceed 1%. The latter suggests that the model has a nearly absolute convergence in terms of the average stress. For this reason, we focused on smoothness of the phase boundary description (see Figure 5) and coincidence of the major strain localization patterns. Based on the convergence verification according to these two parameters, a smooth description of a phase boundary is achieved when the number of mesh elements exceeds 20 mln. Meanwhile, the major strain localization pattern ceases to change when the number of mesh elements exceeds 22 mln. Therefore, the mesh with ≈22 mln elements was chosen as a reasonable trade-off between the accuracy and the computational costs. Influence of pores array on the results of model estimations is discussed in Appendix A.

### 5.2. Stages of Deformation Process

**Figure 6:**
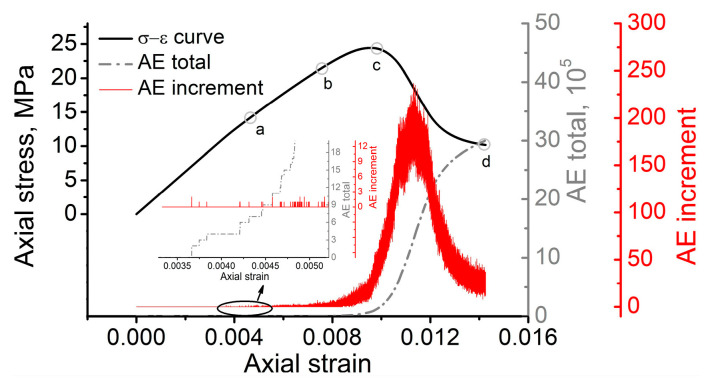
Stress-strain curve combined with simulated acoustic emission pulses.

Let us now carefully trace the stress-strain evolution in the course of sample loading. The major interest is focused on the stages of sample deformation, since they outline the threshold stresses of crack initiation, crack damage and peak in the loading diagram. To obtain these values, we used several approaches based on the simulations of acoustic emission (AE) pulses, instantaneous Poisson’s ratios, and the reversal point method. The simulation of AE is related to the interpretation of a number of elements which experienced complete damage, i.e., either parameter D reached unity or parameter Y became zero due to strain softening. In each time step of numerical integration, we calculated the number of elements which had met the condition discussed in the previous sentence. Further, we combined this temporal diagram with the stress-strain curve of the loaded sample to understand the regularity of fracture process scaling. Figure 6 illustrates the stress-strain curve of the loaded sample combined with the simulated AE pulses. The grey curve represents the total accumulated number of pulses, and the red curve represents the increment of the simulated AE pulses.

The experimental findings reported elsewhere [5,37,38] suggest that in the case of uniaxial compression loading the first AE pulses associated with the initiation of new cracks are observed at an axial stress in the range of 40–60% of $\sigma_{p}$. Based on the experimental results obtained in this work, the crack initiation threshold stress of the considered rock-like material falls into the reported range and equals to ≈53%. The numerical modeling stress-strain curve and the simulated AE pulses suggest that departure from linearity and the first pulses occur when the average axial stress level reaches ≈12 MPa, which is ≈57% of peak stress $\sigma_{p}$. Therefore, the model predicts the crack initiation stress threshold with less than 10% error, which is quite reliable.

**Figure 7:**
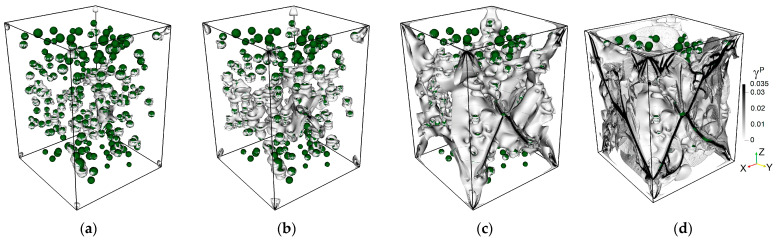
Stages of inelastic strain accumulation related to the corresponding states (**a**–**d**) in Figure 6. The color legend and orientations are the same as in state (**d**). Accumulated damage is represented in the bulk of the sample (grey and black colors) and is combined with the pore structure (pores are shown by dark green color).

**Figure 8:**
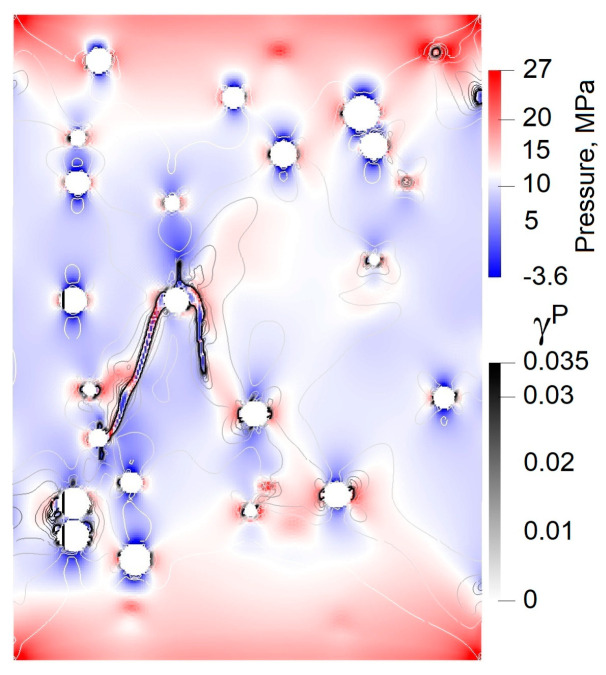
Combined pattern of hydrostatic pressure and inelastic strain in vertical cross-section of the model slightly prior to state (c).

State (a) in the simulated stress-strain curve is associated with the crack initiation stress threshold ($\sigma_{ci}$). Figure 7 illustrates the patterns of accumulated damage in the consecutive instants a–d corresponding to the states marked in the loading diagram a–d in Figure 6. It can be noted that nucleation of strain localization is attributed to the phase boundary wherein the stress concentration is observed (see Figure 8). We can also note that mode I or tensile cracks nucleate in the regions of bulk tension (hydrostatic pressure P<0), while mode II or shear cracks tend to nucleate in the regions of bulk compression (hydrostatic pressure P>0).

From state (a) to state (b) we can see an insufficient increase in strain localization due to a stable growth of the damage degree. In this stage of loading, the number of stress concentration zones exhibit a slight increase due to the energy supply associated with continuing loading. The same is also observed for the increment of AE pulses.

**Figure 9:**
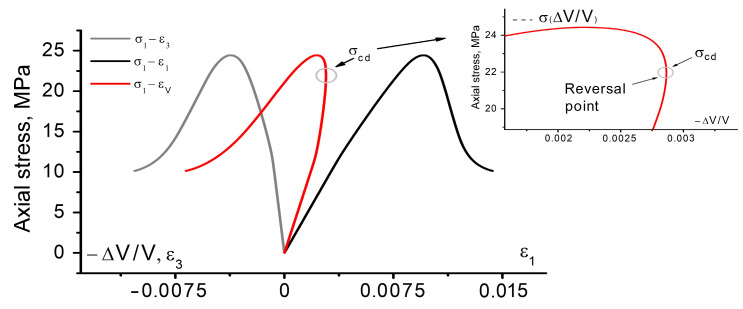
Complete stress-strain curve obtained by numerical modeling.

The results of numerical modeling suggest that we are unable to accurately identify the exact value of $\sigma_{cd}$ based on the AE pulse diagram. To do so, we need to combine the simulated AE curve with the instantaneous Poisson’s ratio and the reversal point method. Let us consider the curves of dependence of the instantaneous Poisson’s ratio calculated as $\mu_{31}=-\varepsilon_{3}/\varepsilon_{1}$, the bulk strain ΔV/V versus the axial strain $\varepsilon_{1}$, and the complete stress-strain curve obtained by numerical modeling. Figure 9 illustrates the complete stress-strain curve obtained by numerical modeling, which was further used to determine the crack damage threshold stress.

**Figure 10:**
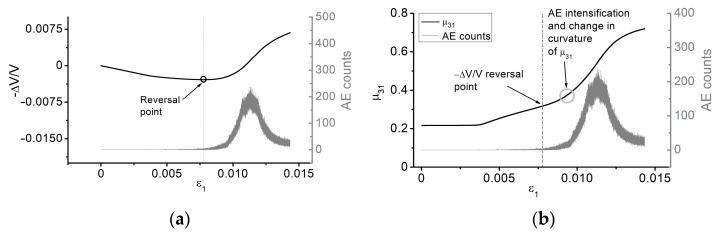
Curves of dependence combined with AE increment: (**a**) volumetric strain, (**b**) instantaneous Poisson’s ratio.

Processing the complete stress-strain curve allowed us to obtain the curves of dependence of $\mu_{31}(\varepsilon_{1})$ (see Figure 10a) and $\Delta V/V(\varepsilon_{1})$ (see Figure 10b). When the derivative of the $\Delta V/V(\varepsilon_{1})$ function changes its sign, the axial stress level is ≈22 MPa. Therefore, the ratio between the crack damage threshold $\sigma_{cd}$ determined according to the reversal point method and the peak stress $\sigma_{p}$ is equal to 0.89, which falls well into the range reported by Hoek and Martin for rocks [5] and satisfactorily meets the experimental data obtained in this work. Notably, a change in the curvature of $\mu_{31}(\varepsilon_{1})$ and a significant intensification of AE are observed at an axial stress slightly above the value obtained by the reversal point method. Moreover, the choice of the crack damage threshold based on these two methods is rather voluntary in contrast to the strict reversal point method. Note that these methods for determining the crack damage threshold can be harmonized with each other if the following rule of thumb is used. When the average number of AE pulses per unit time exceeds the emission in the initial stage of crack initiation by $e^{2}$ times, a transition to the stage of unstable crack formation is observed.

As the crack damage stress threshold ($\sigma_{cd}$) is approached, the increment of the simulated AE pulses continuously increases. Finally, the onset of the unstable deformation stage becomes pronounced (after state (b)), which is associated with the explosive increase in the number of AE pulses. Notably, the majority of elements exhibiting complete damage is located in the bulk of the sample. The coalescence of these damaged elements into larger zones of strain localization is manifested in the post-peak deformation stage when the stress-strain curve demonstrates a descending character. As the residual strength state (d) is approached (see Figure 7d), we can observe the final fracture pattern of the sample, with the strain localization zones reaching the free surfaces of the sample. One can see that a mixed fracture mechanism is realized, since both the subvertical tensile cracks and inclined shear cracks break the volume of the sample into many fragments. However, shear cracking is obviously dominant, with the inclination angles lying in the range of 45–70° to the loading direction.

## 6. Conclusions

In this work, we have discussed the experimental and computational studies of the stress-strain evolution in sandy-cement samples subjected to uniaxial compression. The experimental results were successfully used as a guide for validation of the model parameters. The model was further used to determine the threshold stresses that are important for distinguishing the deformation process stages. The results obtained might be useful in the engineering practices of rock pressure control. The results of numerical modeling satisfactorily meet the available literature data and sufficiently complement the experimental data.

The following conclusions have been drawn relying on the numerical modeling data:

1. the AE and instantaneous Poisson’s ratio methods are better suited for determination of the crack initiation threshold stress than the crack damage threshold stress; meanwhile, the reversal point method is more suitable for determination of the crack damage threshold stress; it is due to the following: we are unable to determine strictly the crack initiation threshold using the reversal point method since the deviation from linearity in $\Delta V/V(\varepsilon_{1})$ dependence is not instantaneous but occupies some strain range. The AE and instantaneous Poisson’s ratio methods, on a contrary, give a strict value of strain when the first cracks are produced. And vice versa, the reversal point method gives the strict value of crack damage threshold stress in a contrast to the AE and instantaneous Poisson’s ratio methods, which give the strain range of gradual change in curvature of AE rate as a function of $\varepsilon_{1}$ and $\mu_{31}(\varepsilon_{1})$.
2. the stages of deformation in the loading diagrams are strongly related to the sample damage evolution; in other words, the sample is able to resist loading as long as the areas of localized damage grow steadily and do not interact with each other. When the areas of localized damage begin to interact with each other, the stage of unstable avalanche growth of damage and loss of bearing capacity (global strength) begins.
3. the formation of tensile and shear cracks is associated with the structural inhomogeneities—pores in the case under study as regions of strong stress concentration;
4. the model provides a reliable basis for simulating the regularities of AE and the corresponding damage, as well as the major failure patterns of the samples. It is shown that the patterns of damage evolution qualitatively correlate with the patterns of acoustic emission evolution under uniaxial loading of rock samples. The merging of localized damage regions of smaller scales forms a macroscopic failure pattern, which demonstrates the possibility of describing mixed failure mechanisms of loaded rocks and correlates with experimental data.

## Figures and Tables

**Table 1:** Summary of experimental data.

Exp.	w, mm	l, mm	h, mm	E, GPa	ν	$\sigma_{p},\;\mathrm{MPa}$	$\frac{\sigma_{cc}}{\sigma_{p}}$	$\frac{\sigma_{ci}}{\sigma_{p}}$	$\frac{\sigma_{cd}}{\sigma_{p}}$
Red	14.8	15.0	20.6	3.65	0.30	26.0	0.05	0.45	0.88
Green	14.8	14.8	22.2	2.72	0.14	30.4	0.15	0.65	0.86
Blue	14.1	14.5	20.2	4.06	0.27	25.4	0.10	0.48	0.67
Black	14.9	14.9	21.1	3.32	0.24	23.2	0.10	0.63	0.85
Magenta	14.8	14.8	21.2	2.89	0.26	21.2	0.10	0.46	0.66
Violet	14.8	13.8	22.5	2.14	0.13	19.3	0.23	0.48	0.84
Average	14.7	14.6	21.3	3.14	0.24	24.3	0.13	0.53	0.78

**Table 2:** The summary of the model parameters.

$\rho_{0}$ $,\;g/\mathrm{cm}{}^{3}$	K, GPa	μ, GPa	$\sigma_{c},\;\mathrm{MPa}\;$	$\sigma_{t},\;\mathrm{MPa}\;$	$Y_{0},\;\mathrm{MPa}\;$	$\alpha_{1}$	$\alpha_{2}$	$\gamma^{*}$
2.08	2.157	1.26	24.3	2.4	3.82	1.41	0.78	0.003

**Figure A3:**
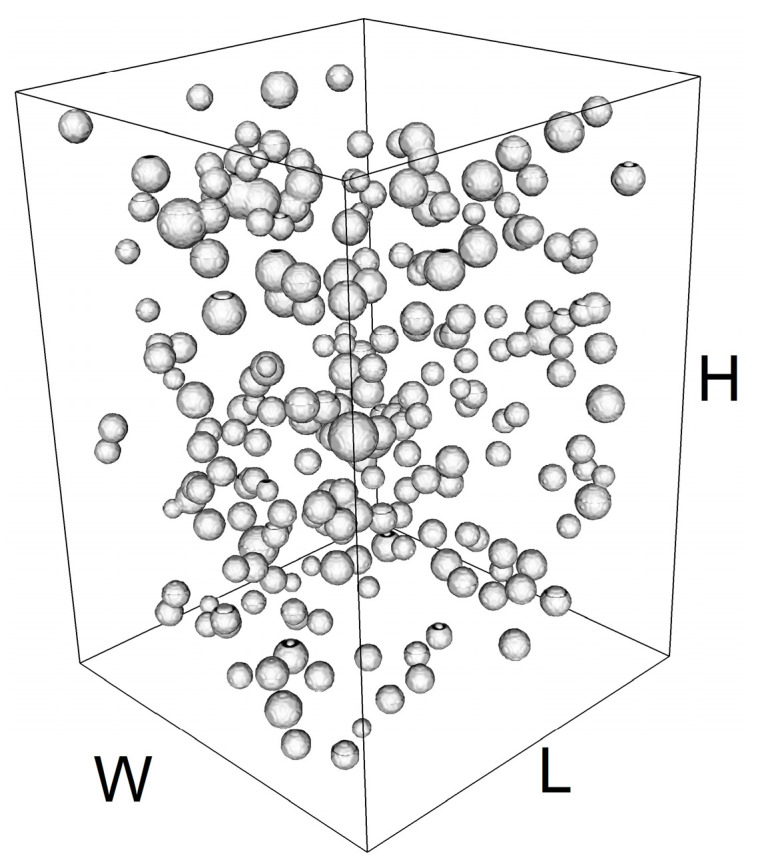
Representative volume element (RVE) of sandy-cement sample. Height of RVE *H* ≈ 715 µm, H:W = 2.1:1.5.

**Figure A4:**
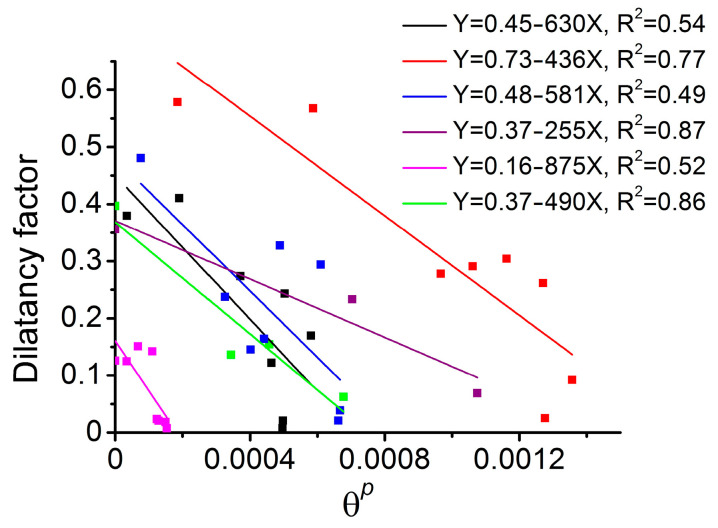
Determination of dilatancy factor curves of dependence on inelastic strain.

**Figure A5:**
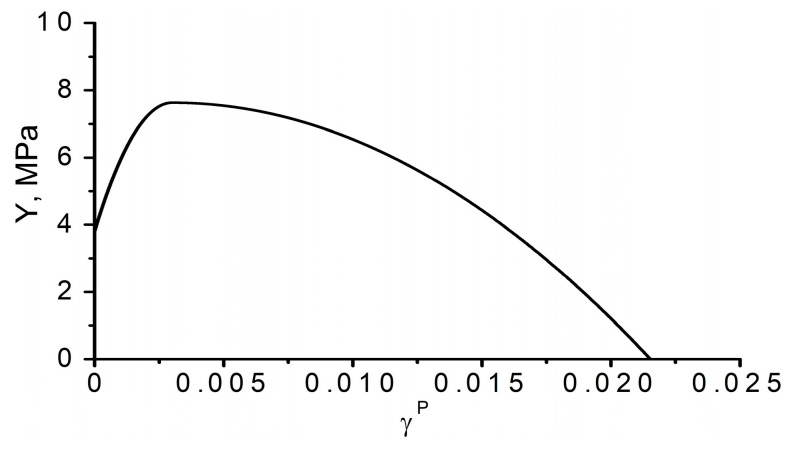
Evolution of cohesion in the course of inelastic strain accumulation.

**Figure A6:**
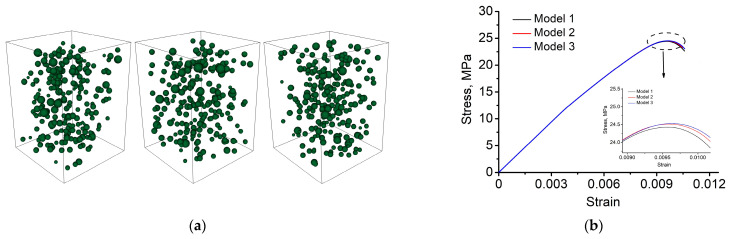
Three different designed structural models with stochastic distribution of pores in computational domain having identical integral porosity (**a**), stress-strain curves obtained for these three models using the same set of parameters (**b**).

## Data Availability

The data presented in this study are available on request from the corresponding author. The data are not publicly available due to being a part of continuing work.

## Appendix A

We used the complete stress-strain curve of the loaded samples to determine the Young’s modulus and the Poisson’s ratio of samples. To do so, we extracted the part of stress-strain curve obeying the Hooke’s law (marked by black lines in both plots). The slope of the $\sigma_{1}-\varepsilon_{1}$ plot and the absolute slope of the $\varepsilon_{3}-\varepsilon_{1}$ curve gave us the Young’s modulus and the Poisson’s ratio, respectively.

We used the pseudo-random number generator to design the structural model of the representative volume element (RVE). The pore nucleation centers were randomly distributed within the computational domain. The pore sizes satisfy the experimental PDF (Figure A2b) and the total porosity of RVE is ≈2%. The aspect ratio W:H:L is kept the same as in the experiment. Note that the pores are explicitly included into the model as definite regions of representative volume element wherein the free-of-stress condition is maintained. It allows not to change the formulation of strain rate Equation (3) since strain rates are calculated only in “matrix”.

The initial value of Y, and the reference values of $\alpha_{1}$ and $\alpha_{2}$ are determined according to Equations (A1)–(A3), respectively.

(A1) $$Y=\sigma_{t}(\frac{1}{\sqrt{3}}+\frac{\alpha_{1}}{3})$$

(A2) $$\alpha_{1}=\sqrt{3}\frac{\sigma_{c}-\sigma_{t}}{\sigma_{c}+\sigma_{t}}$$

(A3) $$\alpha_{2}=\frac{\frac{1}{\sqrt{3}}\sigma_{c}-Y}{\frac{1}{3}\sigma_{c}}$$

Processing of the stress-strain curves for all tested samples allowed us to determine the curves of the dilatancy factor dependence on the bulk inelastic strain. Despite the fact that some approximations have low confidence level (≈0.5), we averaged the slopes and intercepts of all approximations in Figure A4 and used the resulting equation in the model as the zero hypothesis. Note that a non-constant dilatancy factor is a necessary condition for the agreement between the numerical modeling and the experiment. Otherwise, the stress-strain evolution is improperly described, and we observe blurred strain localization pattern with rather thick strain localization. However, the use of experimentally obtained equation for dilatancy factor in a point of continuum in modeling yields insufficient dilatancy which results in a sharp drop of average stress and contradiction with experimental curves. The dilatancy factor equation in a point of continuum which was finally used is given by Equation (A2). Figure A5 illustrates the cohesion evolution curve in a point of continuum.

(A4)
$$\Lambda =0.6-30\theta^{P}$$

### Influence of Pores Array on the Results of Model Estimations

To ensure that the array of pores in the computational domain does not contribute to the model estimates of threshold stresses, we additionally designed two models with different stochastic distributions of pores (Figure A6a). At the same time, the integral porosity and pore size distributions in mesovolumes remained identical. Based on the obtained modeling results, the mechanical response of mesovolumes remained almost identical—the difference in estimated values of threshold stresses is less than 0.5%. Therefore, we can conclude that the array of pores does not contribute to the numerical results in the case of stochastic distribution of pores within the computational domain.

## References

[1] Bieniawski Z.T. (1967). Mechanism of brittle fracture of rock: Part II—Experimental studies. Int. J. Rock Mech. Min. Sci. Geomech. Abst..

[2] Brace W.F., Paulding B.W., Scholz C. (1966). Dilatancy in the fracture of crystalline rocks. J. Geophys. Res..

[3] Cai M., Kaiser P.K., Tasaka Y., Maejima T., Morioka H., Minami M. (2004). Generalized crack initiation and crack damage stress thresholds of brittle rock masses near underground excavations. Int. J. Rock Mech. Min. Sci..

[4] Diederichs M.S. (2007). The 2003 canadian geotechnical colloquium: Mechanistic interpretation and practical application of damage and spalling prediction criteria for deep tunnelling. Can. Geotech. J..

[5] Hoek E., Martin C.D. (2014). Fracture initiation and propagation in intact rock—A review. J. Rock Mech. Geotech. Eng..

[6] Martin C.D., Chandler N.A. (1994). The progressive fracture of lac du bonnet granite. Int. J. Rock Mech. Min. Sci. Geomech. Abst..

[7] Nicksiar M., Martin C.D. (2013). Crack initiation stress in low porosity crystalline and sedimentary rocks. Eng. Geol..

[8] Ji P.-Q., Zhang X.-P., Zhang Q. (2018). A new method to model the non-linear crack closure behavior of rocks under uniaxial compression. Int. J. Rock Mech. Min. Sci..

[9] Peng J., Rong G., Cai M., Zhou C.-B. (2015). A model for characterizing crack closure effect of rocks. Eng. Geol..

[10] Martin C.D., Christiansson R. (2009). Estimating the potential for spalling around a deep nuclear waste repository in crystalline rock. Int. J. Rock Mech. Min. Sci..

[11] Jiang Q., Feng X.-T., Chen J., Huang K., Jiang Y.-L. (2013). Estimating in-situ rock stress from spalling veins: A case study. Eng. Geol..

[12] Esterhuizen G.S., Dolinar D.R., Ellenberger J.L. (2011). Pillar strength in underground stone mines in the united states. Int. J. Rock Mech. Min. Sci..

[13] Xue L., Qin S., Sun Q., Wang Y., Lee L.M., Li W. (2014). A study on crack damage stress thresholds of different rock types based on uniaxial compression tests. Rock Mech. Rock Eng..

[14] Zhao X.G., Cai M., Wang J., Ma L.K. (2013). Damage stress and acoustic emission characteristics of the beishan granite. Int. J. Rock Mech. Min. Sci..

[15] Ghazvinian E., Diederichs M.S., Quey R. (2014). 3d random voronoi grain-based models for simulation of brittle rock damage and fabric-guided micro-fracturing. J. Rock Mech. Geotech. Eng..

[16] Potyondy D.O., Cundall P.A. (2004). A bonded-particle model for rock. Int. J. Rock Mech. Min. Sci..

[17] Sinha S., Walton G. (2020). A study on bonded block model (bbm) complexity for simulation of laboratory-scale stress-strain behavior in granitic rocks. Comput. Geotech..

[18] Zhou J.W., Xu W.Y., Yang X.G. (2010). A microcrack damage model for brittle rocks under uniaxial compression. Mech. Res. Commun..

[19] Liu Z., Zhang C., Zhang C., Gao Y., Zhou H., Chang Z. (2019). Deformation and failure characteristics and fracture evolution of cryptocrystalline basalt. J. Rock Mech. Geotech. Eng..

[20] Wang T., Xu D., Elsworth D., Zhou W. (2016). Distinct element modeling of strength variation in jointed rock masses under uniaxial compression. Geomech. Geophys. Geo-Energy Geo-Resour..

[21] Zhou G.-L., Xu T., Heap M.J., Meredith P.G., Mitchell T.M., Sesnic A.S.-Y., Yuan Y. (2020). A three-dimensional numerical meso-approach to modeling time-independent deformation and fracturing of brittle rocks. Comput. Geotech..

[22] Wang Y., Zhou X., Shou Y. (2017). The modeling of crack propagation and coalescence in rocks under uniaxial compression using the novel conjugated bond-based peridynamics. Int. J. Mech. Sci..

[23] Zhao Y. (1998). Crack pattern evolution and a fractal damage constitutive model for rock. Int. J. Rock Mech. Min. Sci..

[24] Xie N., Zhu Q.Z., Xu L.H., Shao J.F. (2011). A micromechanics-based elastoplastic damage model for quasi-brittle rocks. Comput. Geotech..

[25] Yuan X.P., Liu H.Y., Wang Z.Q. (2013). An interacting crack-mechanics based model for elastoplastic damage model of rock-like materials under compression. Int. J. Rock Mech. Min. Sci..

[26] Lingfan Z., Duoxing Y., Zhonghui C. (2019). Deformation and failure characteristics of weathered granite under uniaxial compression. AIP Adv..

[27] Kostandov Y.A., Medvedev V.S. (2011). Analysis of limiting state of fractured brittle bodies under uniaxial compression. Zavod. Lab..

[28] Atapour H., Mortazavi A. (2018). The influence of mean grain size on unconfined compressive strength of weakly consolidated reservoir sandstones. J. Pet. Sci. Eng..

[29] Farrokhrouz M., Asef M.R. (2017). Experimental investigation for predicting compressive strength of sandstone. J. Nat. Gas Sci. Eng..

[30] Smolin A.Y., Eremina G.M., Korostelev S.Y. (2019). Dependences of mechanical properties of ceramics with bimodal pore size distribution on the porosity at various scale levels. Russ. Phys. J..

[31] Drucker D.C., Prager W. (1952). Soil mechanics and plastic analysis or limit design. Q. Appl. Math..

[32] Stefanov Y.P., Chertov M.A., Aidagulov G.R., Myasnikov A.V. (2011). Dynamics of inelastic deformation of porous rocks and formation of localized compaction zones studied by numerical modeling. J. Mech. Phys. Sol..

[33] Eremin M. (2021). Finite-difference numerical analysis of faulting and accompanying seismicity near the Chuya and Kurai depressions, Gorny Altai, Russia. Tectonophysics.

[34] Makarov P.V., Eremin M.O. (2013). Fracture model of brittle and quasibrittle materials and geomedia. Phys. Mesomech..

[35] Wilkins M.L. (1999). Computer Simulation of Dynamic Phenomena.

[36] Eremin M. (2020). Three-dimensional finite-difference analysis of deformation and failure of weak porous sandstones subjected to uniaxial compression. Int. J. Rock Mech. Min. Sci..

[37] Kong B., Wang E., Li Z., Wang X., Liu J., Li N. (2016). Fracture mechanical behavior of sandstone subjected to high-temperature treatment and its acoustic emission characteristics under uniaxial compression conditions. Rock Mech. Rock Eng..

[38] Kong B., Wang E., Li Z., Wang X., Niu Y., Kong X. (2017). Acoustic emission signals frequency-amplitude characteristics of sandstone after thermal treated under uniaxial compression. J. Appl. Geophys..

